# Letter from France

**Published:** 1881-08

**Authors:** 


### Foreign Correspondence.

---

ARTICLE XI.

VILLERS-SUR-MER,

NORMANDY, FRANCE, June, 1881.

MESSRS. EDITORS :—In crossing the sea with a large party of his own patients, a physician naturally interests himself in the symptoms of, and the effect of treatment upon, that invariable element of an ocean trip, sea-sickness. Dr. Beard, of New York, has lately written a pamphlet upon this complaint, and his chief weapon against its inroads is the potassium bromide, with which he advises physicians to intoxicate their patients before they go on board the steamer. This bromidism is then to be kept up during the passage, and thus, thinks Dr. Beard, sea-sickness may be kept at bay. I regret that I cannot endorse this opinion and this advice. In the first place I can hardly believe that any physician of calm judgment would place delicate women under such overwhelming influence of bromide for several days before they go to sea and continue it during the voyage. Secondly, I do not believe such treatment would overcome or prevent sea-sickness. Given in proper doses, while at sea, the bromides in my hands effected nothing whatever. Nitrate of amyl is another remedy suggested by others as well as by Dr. Beard. I used it in strong doses. It not only did no good whatever, but its penetrating, to many, nauseating, odor disturbed the stomachs of those who otherwise, perhaps, would not have been so ill. Dr. Beard thinks the galvanic current an excellent remedy in sea-sickness, but says its use at sea is impracticable. By cutting small openings in the cover of a Hall's French battery for the passage of the wires and afterward suspending the box, I

found no difficulty whatever in passing the Faradic current through the stomachs of my patients, the positive pole being placed in the epigastric region, the negative over the base of the brain. In one case this procedure seemed to effect a temporary relief. In all others it failed to accomplish any good whatsoever. The citrate of coffeine is also recommended. Its effects are *nil.* In short, I believe there are only two remedies for sea-sickness, and they are rather palliative than curative, but they do lessen the duration of the attack. One of these is *fresh air* taken in a supine position on deck, the other is indomitable *pluck.* If patients cannot be kept perfectly warm on deck it is better that they keep their berths. Cold at sea is extremely exhaustive. In one of my patients it caused an attack of neuralgia which exposed the lady to serious danger. Food of simple character, such as biscuit, oatmeal porridge, baked potatoes, well salted, and a little black coffee without sugar, will gradually accustom the stomach to food of more solid character. But the rule should be that if such simple nourishment be ejected by the stomach, a fresh quantity should at once be taken. It is not necessary to say that lemons, oranges (and perhaps apples), are indispensible.

A small mustard plaster over the epigastric region is sometimes a comfort, and hot water at the feet is a delight. But the *medical* treatment of sea-sickness is a snare and a delusion. It not only does no good, but I believe rather increases than assuages that feeling of unspeakable disgust, which led Charles Dickens to say on the first day out, that he was afraid he should die, and on the second, that he was afraid he should not.

In several voyages across the Atlantic I have as yet failed to meet a ship's surgeon who had any faith in medical remedies against sea-sickness. These gentlemen are as heretical in this direction as the veriest old sea-dog before the mast. They try to sympathize with sea-sick passengers but are quite ready to confess their absolute ignorance of effectual remedies. I am inclined to believe, however, that the doctors on ocean steamers are not overwhelmed by ambition. For the most part they are genial, pleasant fellows, ready and willing to do one a kindness. They go back and forth between two ports, year after year, quite satisfied, apparently, with their lot and their salary of four or five hundred dollars. They are generally unmarried. Living costs them nothing. They have a good table, and their principal outlay is for the clothes they wear. So far as I can learn the majority of them are contented, or at any rate feel that they cannot do better than respond to the limited calls upon their professional skill at sea, and therefore do not attempt to secure anything better. It is a pleasure to feel that they do not expect to be feed at the end of the voyage. With the exception of the doctor and the captain and perhaps the first mate, I suppose there is no soul of the service of a sea steamer who will not accept a "tip" of any dimension. This is the one annoying drawback to the pleasure of the voyage. One of our humorous paragraphists recently said: "A first-class way to commit suicide by starvation is to live in a hotel and not fee the waiter." This strictly applies to a sojourn of ten days on an English ocean steamer. And, remember, unless you give at least ten shillings to the steward who has done nothing for you beyond changing your plates at table, he will not only not thank you in the slightest degree, but will become your enemy. It will not do to starve, for you have a European trip in mind, so you give your steward the ten shillings, or a sovereign, and feel disgusted at the mental degradation of English and European menials of every description. You are allowed to breathe in England and on the continent but hardly without a fee.

On this side of the water the American physician rather dreads to purchase medicines. He quickly appreciates the greater elegance of the preparations of leading druggists at home. If he wishes to procure a bottle of quinine pills he finds a rough looking article, moderate in price it is true, but bearing no comparison with our sugar-coated or our beautiful gelatine-covered pill. The English pill is covered with *French chalk*, of all substances. I was glad to find in London, after a prolonged search, the pills of McKesson & Robins. But the indications were that they were not much in demand.

In France a pill is usually coated with silver foil and is quite satisfactory. The general manipulation of the French druggist is much superior to that of the English pharmacist, but an American physician abroad is disappointed by his failure to find the many handsome and neat preparations of the alkaloids which are so abundant in the United States. The cost of all medicines, however, is much lower in Europe than in our own country. For example, I purchased one-grain quinine pills at the rate of seventy-five cents the hundred. In London an ounce of the potassium chlorate may be purchased for five cents.

While in the latter city last month, I had the pleasure of making the acquaintance of Mr. Howard Marsh, an orthopœdic surgeon of great ability, and a gentleman whose charming courtesy I shall not soon forget. Aside from his cases in one of the large city hospitals he has a special hospital for hip-joint cases, supported by charity. Many of his methods of treatment were unique and original. I shall have more to say about them in a future letter. Mr. Marsh is a young man who holds a high place in the esteem of his London confrères, and I am sure will one day win a reputation which will extend beyond his own country.

Mr. William Adams, of London, made many warm friends among our surgeons during the International Medical Congress at Philadelphia, in 1876. During a call upon him he kindly showed me his plaster casts of the contracted finger cases which are beautifully shown in his recent work on this subject. In the billiard room of his house he has a case filled with these casts. They show the hands upon which he operated. Side by side are the deformed and the restored members. His success is very remarkable. His methods are so original and so simple as well, that I would earnestly advise those of your readers who are interested in the treatment of this deformity to purchase and study the Adams' book. It is published in reprint by Mr. Blakiston, of Philadelphia.

During a recent stay of some weeks at Ventnor in the Isle of Wight, I was strikingly reminded of San Remo, on the Mediterranean, a place which stands very high as a resort for consumptives. Ventnor has the same configuration, in a lesser degree, as San Remo. It is packed away at the base of high hills which protect it from northerly and northwesterly winds, and, therefore, possesses a climate which is milder than that of any other locality in England. English physicians send their chest cases to Ventnor for the winter. It offers many of the advantages of towns along the Mediterranean Sea, and is preferable to Nice and Cannes, because less subjected to sudden changes of temperature and to winds which blow across snow mountains.

It is a poem in the beauty of its setting; its rambling, elbow-jagging streets which wander hither and yon at their own sweet will; its charming villas, thatched cottages and lovely gardens. Verdure is so abundant that in a country walk one finds every porch, spire, post, tree, rock and fence buried beneath a deluge of ivy vine. And in the woods the very ground is a mass of ivy leaves. These vines cover and cling to every possible support. Picturesqueness is a word which describes the whole, from the stately hills and the bold cliffs on the shore, to the humble vine-clad cottage of the peasant. I am writing from a charming little town in Normandy. All about me are the same stone farm houses which are so common in Ventnor, with thatched roofs giving a holding place to thick moss, wild flowers and grasses. I am led to imagine, therefore, that the peasant's houses in the Isle of Wight were built after models left in the island by Normans and their descendants. In Ventnor is a noble charity in the shape of the Royal National Hospital for consumptives and diseases of the chest. Dr. Arthur Hill Hassam was its founder. It is supported by voluntary gift and by the small sum of $2.50 per week, which each patient is obliged to pay. The feature which renders this institution unique, is the plan of treating all patients on the "separate system." Every patient has his own chamber. This, of course, is accompanied by the mental advantage of sparing patients the depressing effect of the ward system of treating consumption. A large percentage of patients become restored to health, and the majority improve under the regimen of the hospital. The institution consists of eight blocks of two houses each, standing side by side. In the sleeping story these houses are only one room deep. Each room, therefore, has the same amount of sunlight. They face to the south and look out over an immense garden toward the sea. Patients are kept strictly to certain rules, which comprise directions for the bath, the hours passed in the open air and the diet. The grounds comprise twenty acres. Vegetables and milk are supplied by the farm.

No incurable cases are admitted. One hundred patients is the limit of the number received. Two additional houses, however, will soon be erected. Verandahs run the whole length of the hospital on the lower and second story, those on the ground floor being covered. So that patients may take fresh air even during rainy days. If space allowed, I would gladly give you details of the admirable success of this institution. It is one of the lions of the place, and Great Britain as well as Ventnor is proud of it.

I will ask your readers to bear in mind that Ventnor, Isle of Wight, is a place to which they may safely send those lung and heart cases which bid fair to improve under suitable change of climate. The real benefit to be derived from such change of course consists in the ability of patients to live out of doors several hours daily. To send incurable cases away from the comforts and surroundings of home has always seemed to me a needless cruelty. The cost of living in Ventnor during the months from October to June is quite reasonable. * *

